# A Semi-Supervised Learning Framework for Classifying Colorectal Neoplasia Based on the NICE Classification

**DOI:** 10.1007/s10278-024-01123-9

**Published:** 2024-04-23

**Authors:** Yu Wang, Haoxiang Ni, Jielu Zhou, Lihe Liu, Jiaxi Lin, Minyue Yin, Jingwen Gao, Shiqi Zhu, Qi Yin, Jinzhou Zhu, Rui Li

**Affiliations:** 1 Department of Hepatobiliary Surgery, Jintan Affiliated Hospital of Jiangsu University, Changzhou, Jiangsu 213200 China; 2 Department of Gastroenterology, The First Affiliated Hospital of Soochow University, # 899 Pinghai St., Suzhou, Jiangsu 215006 China; 3 Suzhou Clinical Center of Digestive Disease, Suzhou, Jiangsu 215006 China; 4 Department of Geriatrics, Kowloon Affiliated Hospital of Shanghai Jiao Tong University, Suzhou, Jiangsu 215006 China; 5 Department of Gastroenterology, Beijing Friendship Hospital, Capital Medical University, Beijing, 100050 China; 6 National Clinical Research Center for Digestive Disease, Beijing Digestive Disease Center, State Key Laboratory of Digestive Health, Beijing, 100050 China; 7 Department of Anesthesiology, Jintan Affiliated Hospital of Jiangsu University, Changzhou, Jiangsu 213200 China; 8 Key Laboratory of Hepatosplenic Surgery, Ministry of Education, The First Affiliated Hospital of Harbin Medical University, Harbin, 150001 China

**Keywords:** Self-supervised learning (SSL), Simple framework for contrastive learning of visual representations (SimCLR), NBI International Colorectal Endoscopic (NICE) classification, Colorectal, Computer-aided diagnosis, Deep learning, Supervised learning, Grad-CAM, t-SNE

## Abstract

Labelling medical images is an arduous and costly task that necessitates clinical expertise and large numbers of qualified images. Insufficient samples can lead to underfitting during training and poor performance of supervised learning models. In this study, we aim to develop a SimCLR-based semi-supervised learning framework to classify colorectal neoplasia based on the NICE classification. First, the proposed framework was trained under self-supervised learning using a large unlabelled dataset; subsequently, it was fine-tuned on a limited labelled dataset based on the NICE classification. The model was evaluated on an independent dataset and compared with models based on supervised transfer learning and endoscopists using accuracy, Matthew’s correlation coefficient (MCC), and Cohen’s kappa. Finally, Grad-CAM and t-SNE were applied to visualize the models’ interpretations. A ResNet-backboned SimCLR model (accuracy of 0.908, MCC of 0.862, and Cohen’s kappa of 0.896) outperformed supervised transfer learning-based models (means: 0.803, 0.698, and 0.742) and junior endoscopists (0.816, 0.724, and 0.863), while performing only slightly worse than senior endoscopists (0.916, 0.875, and 0.944). Moreover, t-SNE showed a better clustering of ternary samples through self-supervised learning in SimCLR than through supervised transfer learning. Compared with traditional supervised learning, semi-supervised learning enables deep learning models to achieve improved performance with limited labelled endoscopic images.

**Supplementary Information:**

The online version contains supplementary material available at 10.1007/s10278-024-01123-9.

## Introduction

The impressive ability of deep learning to learn diverse tasks from vast data sources has greatly contributed to prolific advancements in modern computer vision [1]. The generation of big data in scientific fields has coincided with this growth. Through the utilization of vast datasets, computer vision models have acquired a variety of pattern recognition capabilities, ranging from diagnostics at the physician level to medical scene perception [2].

In contrast to traditional computer-aided diagnostic tools that rely heavily on supervised learning, recent advancements in self-supervised learning (SSL) have paved the way for reducing the dependence on large and annotated datasets [3, 4]. This innovative approach leverages unlabelled data to pretrain models, thus mitigating the need for extensive labelled data. In the field of medical image analysis, SSL has emerged as a promising technique, particularly for identifying complex features and lesions in medical images that require specialized expertise [5]. One prominent SSL approach that has gained significant traction in medical image analysis research is contrastive learning [6]. In 2020, Chen et al. introduced a simple framework for contrastive learning of visual representations (SimCLR) [7]. By incorporating data augmentation techniques and larger batch sizes, SimCLR has achieved remarkable performance among a variety of contrastive learning methods [8, 9].

Labelling endoscopic images is an arduous and costly task that necessitates clinical expertise and a substantial number of qualified images. Insufficient samples can lead to underfitting during training and subsequently cause supervised learning models to perform poorly. Thus, SSL methods, e.g., SimCLR, may offer a promising solution to the challenges posed by limited labelled endoscopic images in computer-aided diagnostic tools [10].

In the field of endoscopy, the NBI International Colorectal Endoscopic (NICE) classification system plays a pivotal role in categorizing colorectal neoplasia based on lesion color, vascular pattern, and surface structure of the mucous membrane [11]. This internationally recognized classification divides colorectal neoplasms into three distinct types, as shown in Supplementary Table 1. Type I: The lesion’s color is similar to or lighter than the surrounding mucosa, with a lack of blood vessels or only sparse, thread-like vessels present, and the surface pattern consists of uniform dots. Type II: The color tends toward brown, with thickened brown vessels surrounding white structures, and the white surface patterns are oval, tubular, or branched. Type III: The color is brown or dark brown, sometimes accompanied by patchy white areas, with some regions showing clearly irregular or absent vessels, and the surface structure is irregular or absent. Correct labelling based on the NICE classification requires the judgement and experience of endoscopists under NBI colonoscopy, instead of confirmation by histological or pathological [12]. The available correctly labelled data sources are limited, which makes training a practicable classification model with good generalizability via supervised transfer learning difficult [13].

Thus, in this study, we aimed to develop a semi-supervised learning framework (SimCLR) to classify colorectal neoplasia based on the NICE classification using narrow-band imaging (NBI) colonoscopic images. First, the proposed framework was trained under SSL using a large unlabelled dataset concerning NBI endoscopic images of colorectal neoplasia; subsequently, it was fine-tuned on a limited dataset of the target task, i.e., the NICE classification; and finally, the proposed framework was evaluated on an independent dataset and compared with models based on supervised transfer learning and endoscopists.

## Methods

### The Proposed Semi-Supervised Learning Framework (SimCLR)

**Fig. 1:**
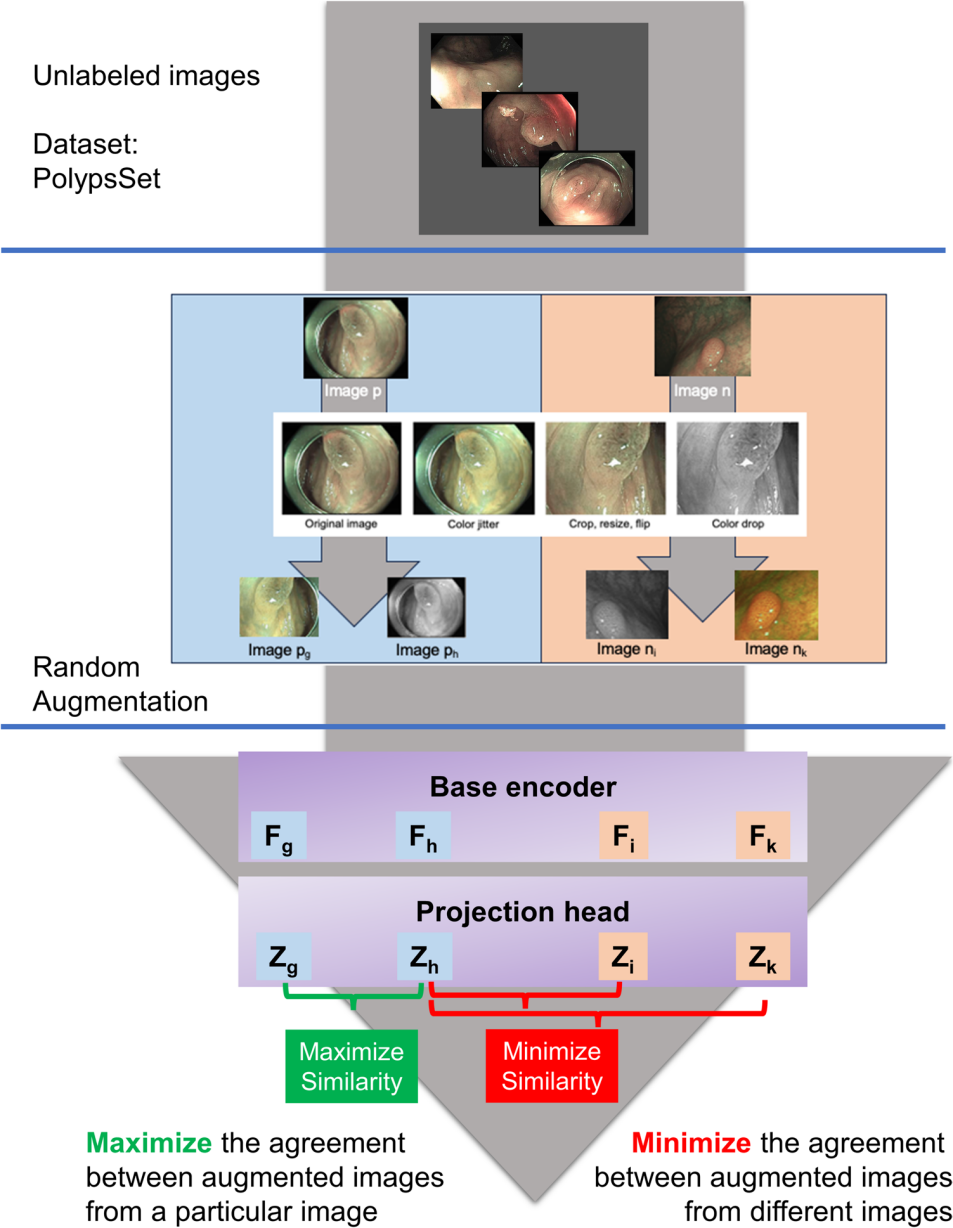
The self-supervised learning (SSL) flowchart

**Fig. 2:**
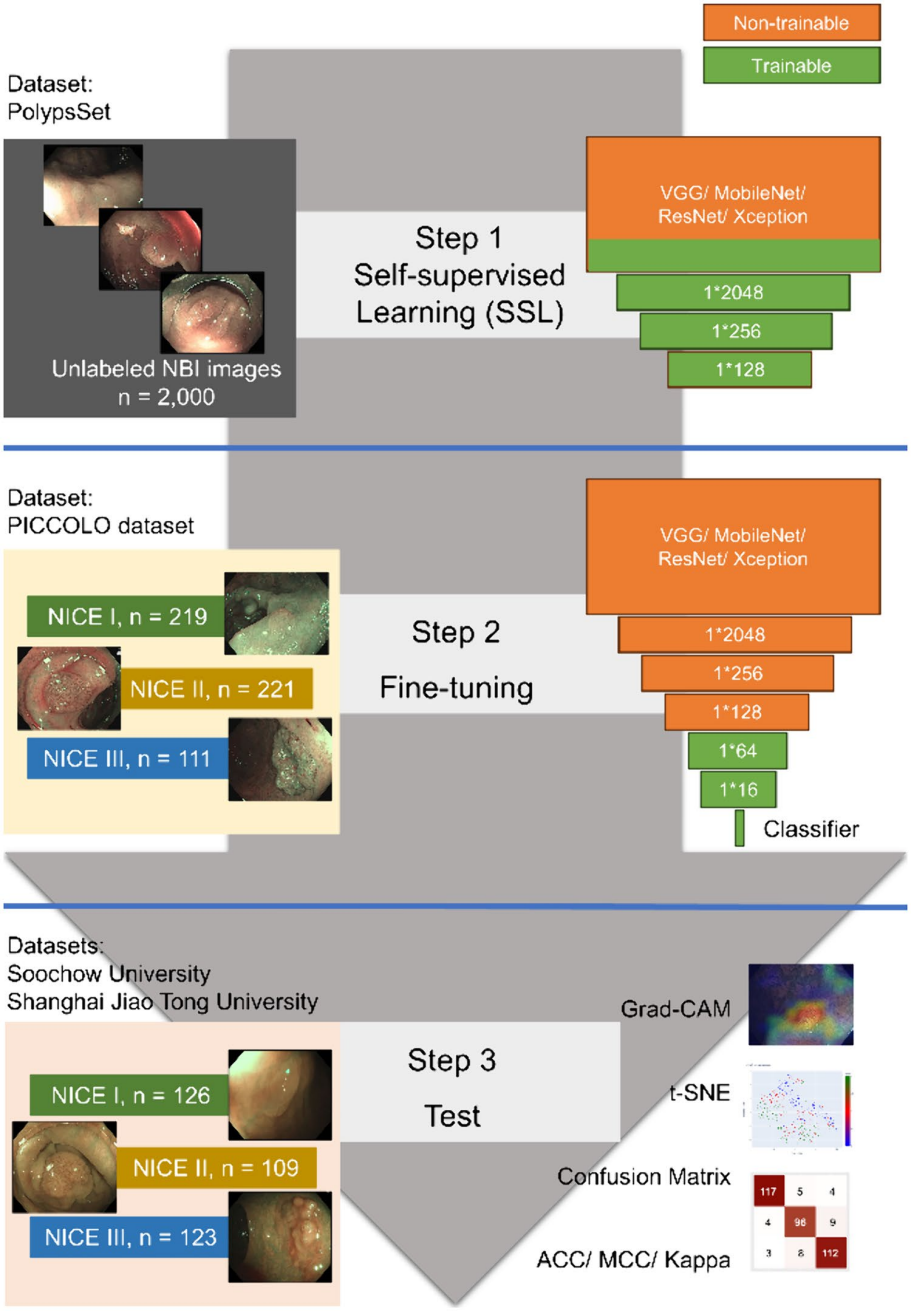
The semi-supervised learning framework (SimCLR). The framework includes two parts: self-supervised learning (SSL) and fine-tuning. The SimCLR-based models were evaluated on the test dataset

The proposed framework, which consists of SSL (Fig. 1) and fine-tuning, is presented in Fig. 2.

### Self-Supervised Learning (SSL) (Fig. 1)

The self-supervised learning model consisted of 3 main components: data augmentation, base encoder, and projection head.

Data augmentation includes a combination of policy, including cropping, flipping, and color distortions. The augmentation module transforms any given data example randomly, resulting in correlated views from a particular image, e.g., two views, denoted as *p*_*g*_ vs. *p*_*h*_, which is considered a positive pair. The module also randomly yields uncorrelated views from different images, i.e., *p*_*h*_ vs. *n*_*i*_ and *p*_*h*_ vs. *n*_*k*_, which are negative pairs [14].

A base encoder is used to extract representation vectors from augmented data examples. The framework allows various selections of the network architecture without any constraints.

Projection head maps representations to the space where contrastive loss is applied.

In our study, unlabelled NBI endoscopic images of colorectal neoplasia from PolypsSet [15] were used to train the SSL model. VGG16, MoblieNet, Resnet50, and Xception (initially trained on ImageNet) were loaded as the backbones of base encoders. We manually removed heads and added three fully connected layers (1*2048, 1*256, and 1*128) to the above backbones. In the SSL procedure, the last three convolutional layers and the three added fully connected layers were trainable, while the other layers of the backbones were nontrainable.

### Fine-Tuning (Fig. 2)

After the initial training using the SSL model, the semi-supervised model was fine-tuned with the labelled NBI images of colorectal neoplasia from the PICCOLO dataset. Two extra fully connected layers (1*64 and 1*16) and a classifier were added. In the fine-tuning procedure, only the two newly added layers and the classifier were trainable for the target task of classification, while the others were nontrainable.

### Supervised Transfer Learning (Fig. 3)

**Fig. 3:**
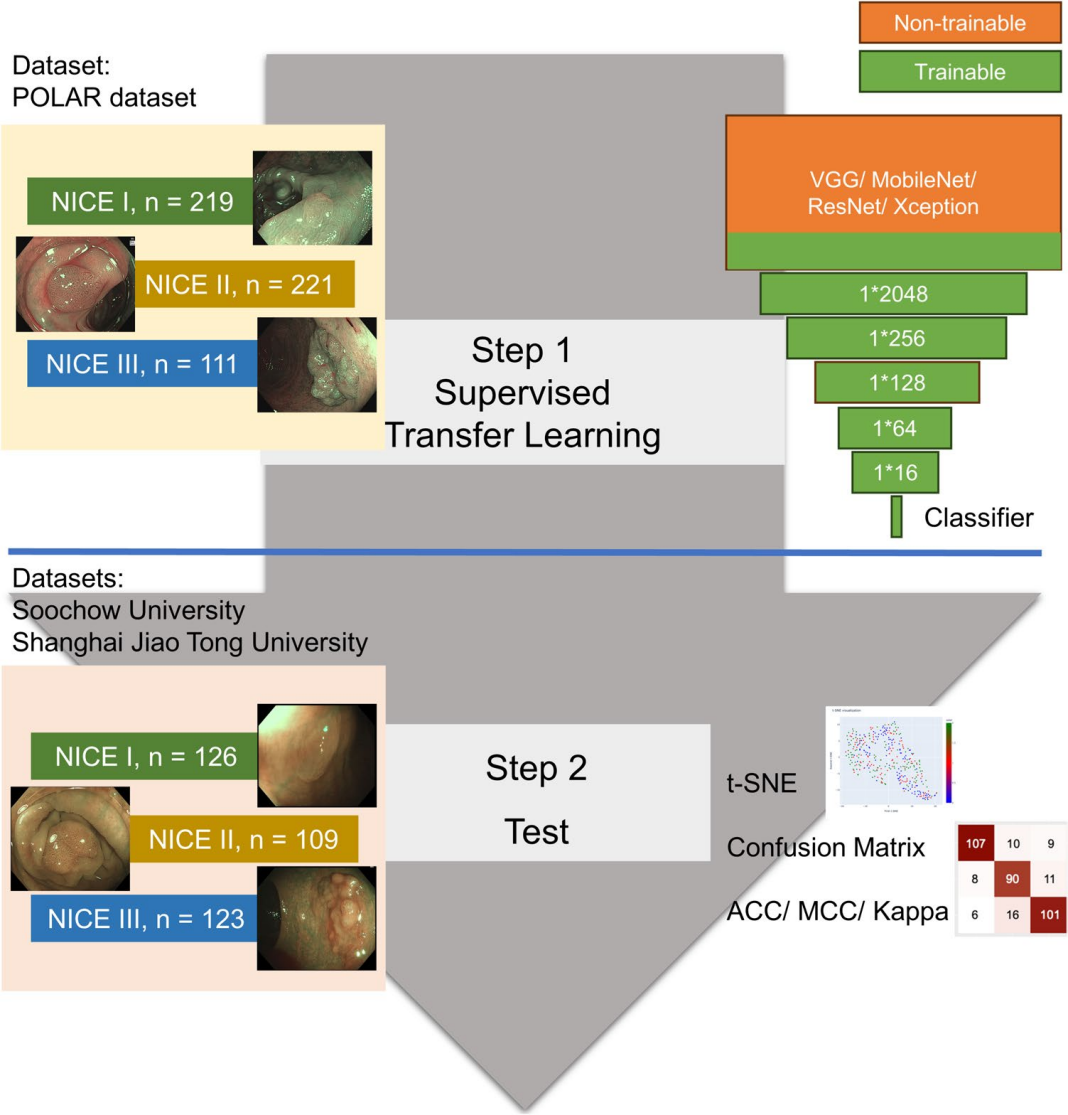
The supervised transfer-learning flowchart

Supervised transfer learning was performed on the dataset as fine-tuning, i.e., the PICCOLO dataset. Like in the case of SSL, four other networks pretrained on ImageNet were loaded. Similarly, five fully connected layers (1*2048, 1*256, 1*128, 1*64, and 1*16) and a classifier were added to the backbones without a head. In the supervised transfer learning procedure, the last three convolutional layers, the five added fully connected layers, and the classifier were trainable, while the others were nontrainable.

### Model Training

The Keras Python (version 3.8.0) platform (backbone: TensorFlow version 2.8.0) was used to train the models. Each image was resized to 224 × 224 pixels and input into the models in the form of RGB channels. The training parameters are listed in Supplementary Table 2. The training code for SSL was inspired by that of Sayak Paul, which is available at https://github.com/sayakpaul/SimCLR-in-TensorFlow-2. Our training code is available at https://osf.io/t3g8n.

### Datasets

#### PolypsSet

Li et al. [15] collected various publicly available endoscopic datasets and a new dataset from the University of Kansas Medical Center to develop a relatively large endoscopic dataset for polyp detection and classification (https://doi.org/10.7910/DVN/FCBUOR). The publicly available dataset includes 155 colorectal video sequences with 37,888 frames from the MICCAI 2017, CVC colon DB, and GLRC datasets [16]. NBI images were collected from the dataset to train the SSL model. To prevent duplication and ensure image quality, three endoscopists with more than 10 years of experience from Soochow University reviewed and finally selected 2000 unlabelled NBI images.

#### PICCOLO

This dataset contains 3433 images from clinical colonoscopy videos, including 2131 white light images and 1302 NBI images, from colonoscopy procedures in 40 human patients (https://www.biobancovasco.bioef.eus/en/Sample-and-data-catalog/Databases/PD178-PICCOLO-EN.html, Basque Biobank: https://labur.eus/EzJUN) [17]. To prevent duplication and ensure image quality, three endoscopists above reviewed and labelled 551 eligible NBI images based on the NICE classification (NICE I, *n* = 219; NICE II, *n* = 221; NICE III, *n* = 111). The labelled 551 endoscopic images were used to fine-tune the semi-supervised model. The detailed information on the two public datasets is presented in Supplementary Table 3.

#### Soochow University/Shanghai Jiao Tong University Dataset

A total of 1432 NBI images of colorectal neoplasia were collected from the First Affiliated Hospital of Soochow University and Kowloon Hospital of Shanghai Jiao Tong University. Three senior endoscopists independently reviewed and labelled 358 eligible images based on the NICE classification (NICE I, *n* = 126; NICE II, *n* = 109; NICE III, *n* = 123). The method for endoscopist reviewing and labelling is shown in Supplementary Fig. 1. The characteristics of colorectal neoplasia are listed in Supplementary Table 4. The dataset was used as an external test dataset. This study was approved by the ethics committee of the First Affiliated Hospital of Soochow University (approval number 2022098).

### Human Endoscopists

To further evaluate the performances of the models, images from the test dataset (Soochow University/Shanghai Jiao Tong University) were evaluated by two independent endoscopists (junior, 3 years of endoscopic experience, and senior, more than 10 years of experience). They had not participated in reviewing or labelling the training images beforehand and were blind to the test set images. They were given 551 labelled images as a reference, then classified the 358 testing images independently. Moreover, to simulate a real clinical environment, two endoscopists were asked to complete the classification assignment within 1 h. A custom web interface was constructed to allow the reviewers to window, zoom, manipulate, and categorize each image.

### Statistical Analysis

A confusion matrix was constructed and used to evaluate the performances of the models and endoscopists. TN, FN, TP, and FP indicate true negatives, false negatives, true positives, and false positives, respectively.

The accuracy represents the proportion of samples that were classified correctly among all samples.

The Matthew correlation coefficient (MCC) [18] measures the differences between the actual and predicted values. The MCC is the best single-value classification metric for summarizing the confusion matrix.

Cohen’s kappa [19] was used to measure the level of agreement between two raters or judges who each classified items into mutually exclusive categories.

A detailed explanation of MCC and Cohen’s Kappa is presented in the Supplementary Introduction.

### Interpretation of Models

#### t-SNE Analysis

In this study, clustering patterns of predictions generated by models were visualized using t-SNE, an unsupervised technique for reducing the dimensionality of data [20]. By leveraging t-SNE with principal component analysis initialization, the high-dimensional vectors were processed and transformed into a two-dimensional visualization, revealing both the local structure and global geometry.

#### Grad-CAM

To enhance the interpretability of convolutional neural networks, Grad-CAM selectively highlights regions in input images that significantly contribute to prediction [21]. This technique could provide insights into how networks make decisions.

A detailed explanation of t-SNE and Grad-CAM is presented in the Supplementary Introduction.

## Results

### Performance of the Models

The four proposed semi-supervised learning (SimCLR) models with various backbones (VGG16, MobileNet, ResNet, and Xception), as well as models based on supervised transfer learning, were developed on the ternary task of the NICE classification. The reason for choosing the above four backbones is presented in the Supplementary Introduction. The performances of the eight models on the external test set are shown in Table 1.

**Table 1:** The classification performance of models and endoscopists using the Soochow University/Shanghai Jiao Tong University dataset

Models/endoscopists		Accuracy	MCC	Cohen’s kappa
Semi-supervised learning (SimCLR)	VGG16	0.844	0.766	0.801 [0.739–0.862]
	MobileNet	0.872	0.808	0.833 [0.788–0.894]
	ResNet	0.908	0.862	0.896 [0.857–0.945]
	Xception	0.885	0.828	0.875 [0.825–0.924]
	Mean	0.875	0.816	0.851 [0.801–0.926]
Supervised transfer learning	VGG16	0.760	0.642	0.677 [0.593–0.746]
	MobileNet	0.799	0.669	0.722 [0.658–0.799]
	ResNet	0.832	0.749	0.790 [0.720–0.851]
	Xception	0.821	0.732	0.779 [0.712–0.841]
	Mean	0.803	0.698	0.742 [0.647–0.819]
Junior endoscopist		0.816	0.724	0.863 [0.828–0.893]
Senior endoscopist		0.916	0.875	0.944 [0.927–0.965]

*MCC* Matthew’s correlation coefficient, *Cohen’s kappa* Cohen’s kappa

**Fig. 4:**
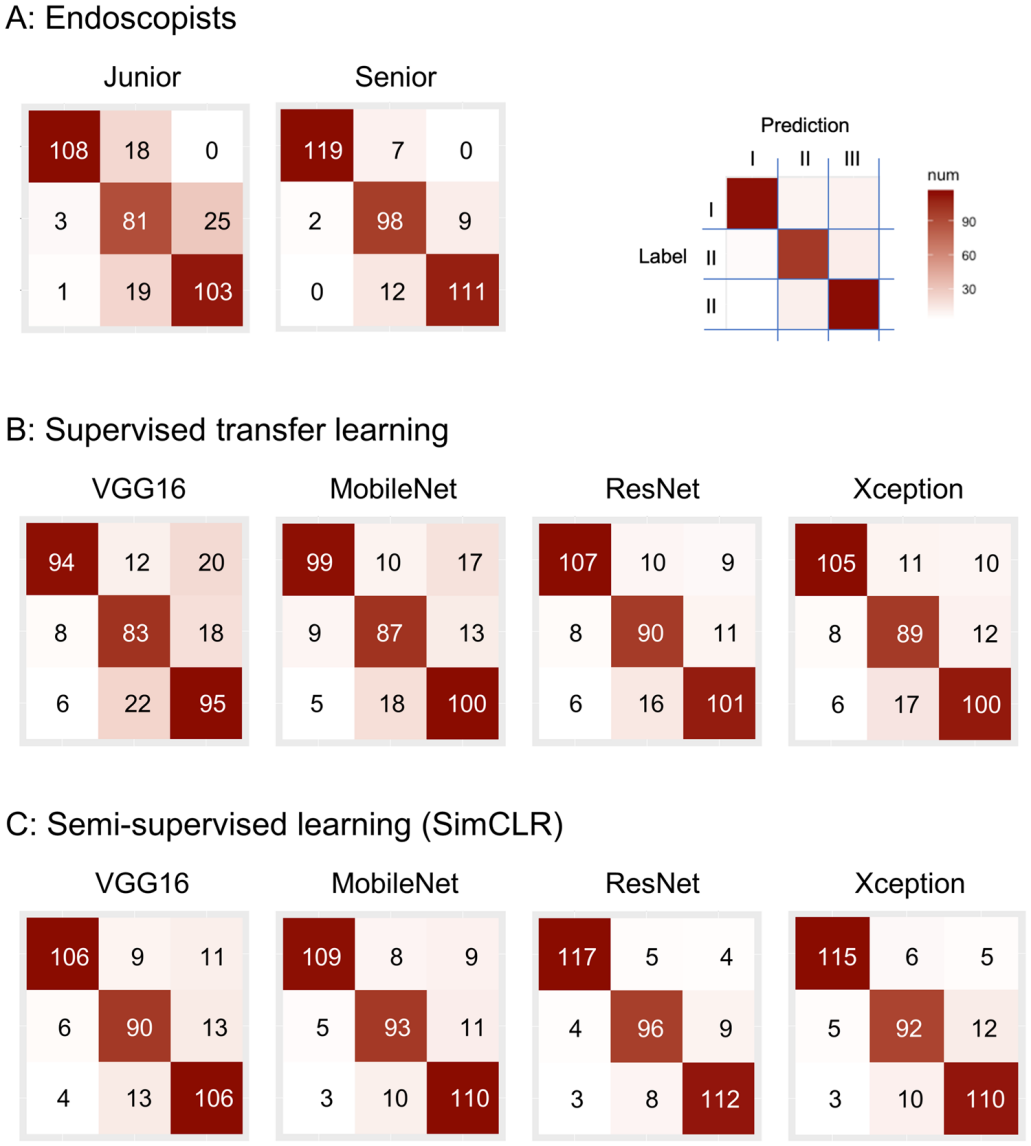
The confusion matrices of the models and endoscopists in the test dataset

Among the models, SimCLR-ResNet achieved the highest accuracy (0.908), followed by SimCLR-Xception (0.885), and SimCLR-MobileNet (0.872). The MCC and Cohen’s kappa of SimCLR-ResNet were 0.862 and 0.896 [0.857–0.945], respectively, which were greater than those of the other models. The confusion matrices are plotted in Fig. 4.

### Comparison with Endoscopists

The performances of the junior and senior endoscopists are listed in Table 1, and their confusion matrices are provided in Fig. 4. The senior radiologist had a higher accuracy, MCC, and Cohen’s kappa coefficient (0.916, 0.875, and 0.944 [0.927–0.965], respectively, than did the SimCLR-ResNet. The junior radiologist had an accuracy, MCC, and Cohen’s kappa of 0.816, 0.724, and 0.863 [0.828–0.893], respectively, which are lower than those of the four SimCLR models.

### Visualized Interpretation of the Models

**Fig. 5:**
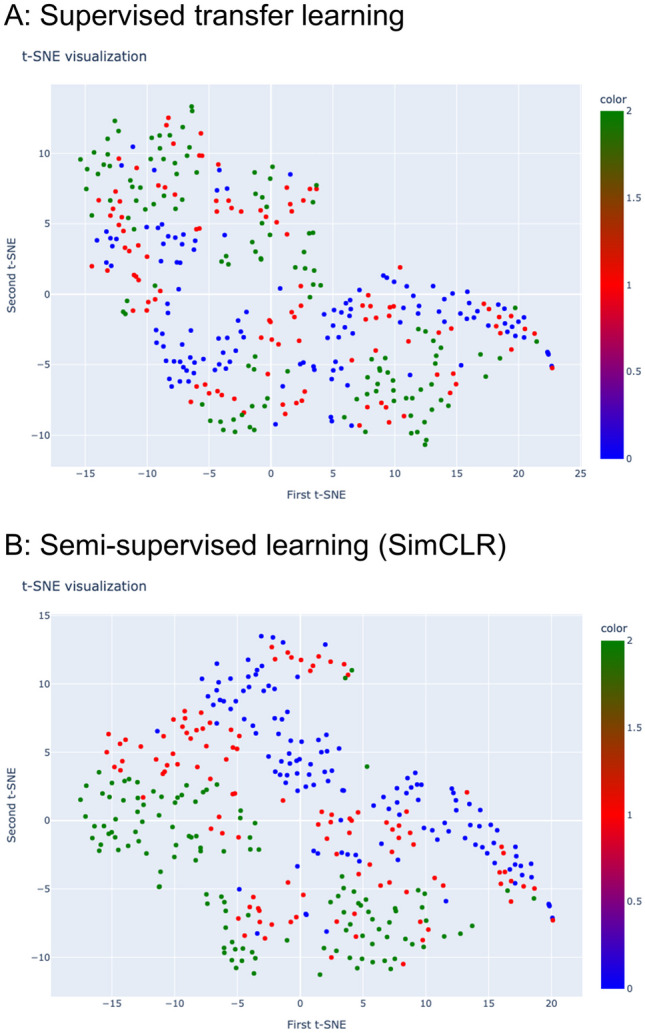
t-SNE visualization of embedded features in the test dataset. **A** Supervised transfer learning: most of the points on the edge of the cluster overlap, and the boundary is not clear. **B** SimCLR: the separation of the three clusters is obviously better than that of part **A**

**Fig. 6:**
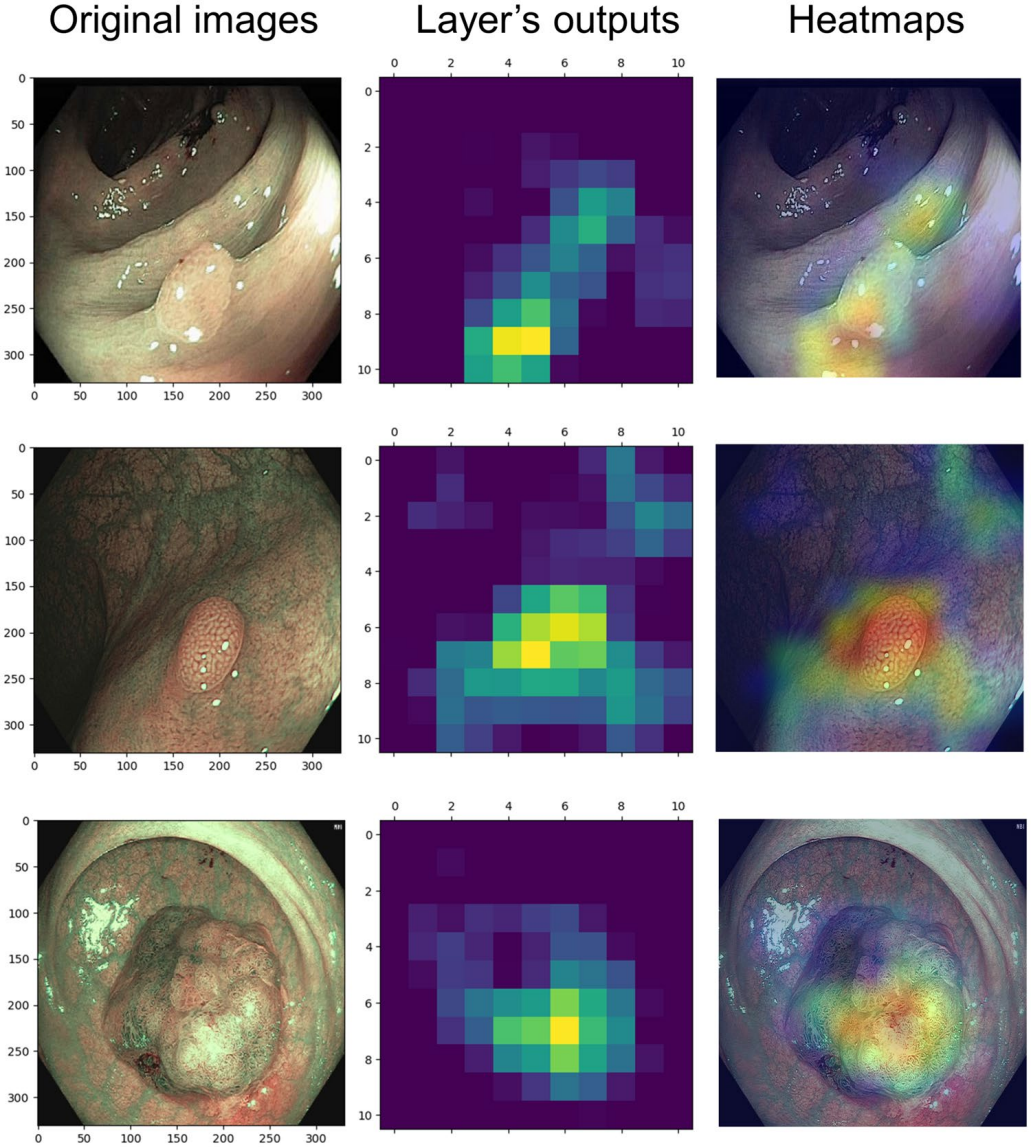
Visualization of SimCLR-ResNet inference via Grad-CAM (the correct examples). The left column: the original endoscopic images. The middle column: heatmaps based on the output of the feature extractor’s last layer of SimCLR-ResNet. The right column: the Grad-CAM heatmap covering the original images, highlighting inferential explanations of the model

**Fig. 7:**
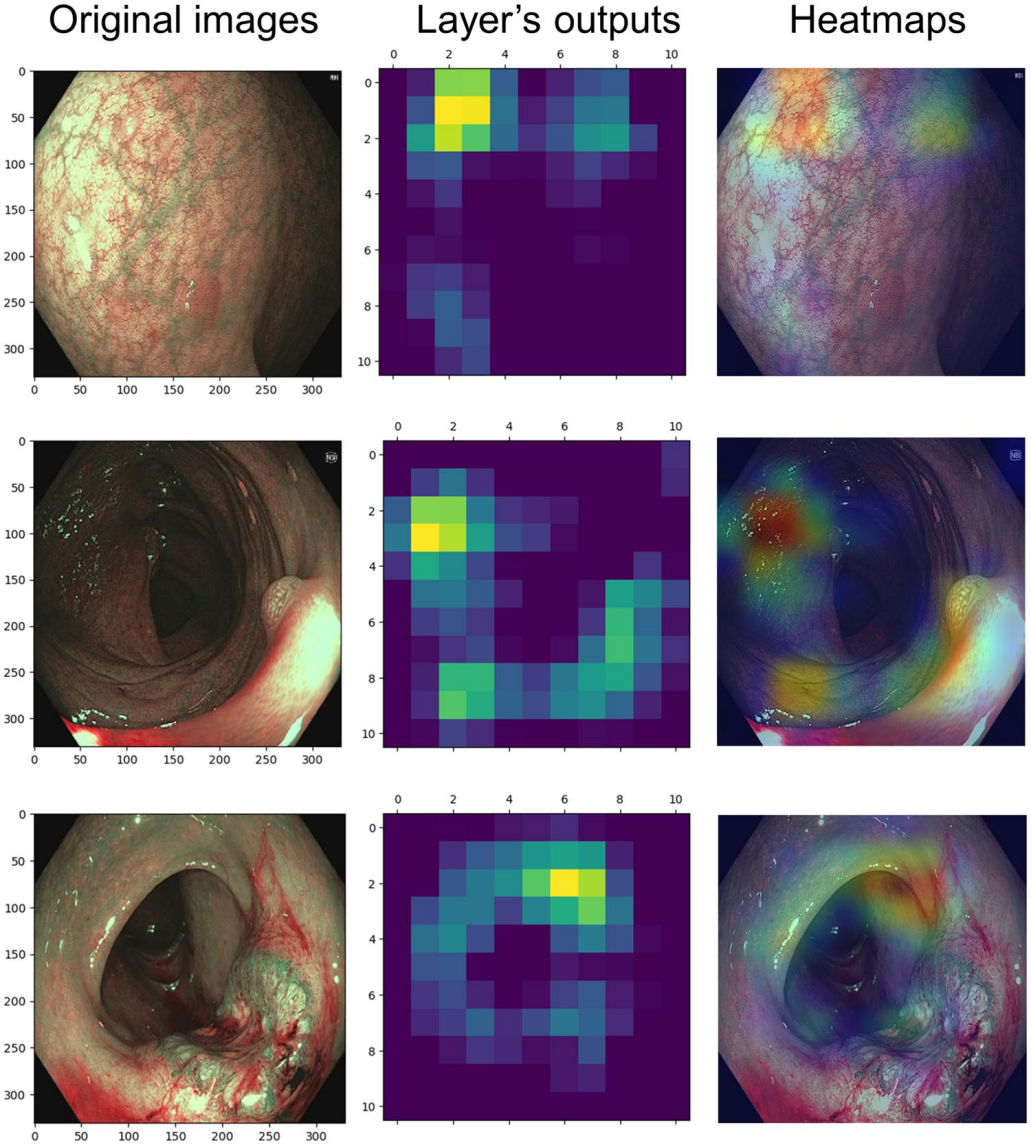
Visualization of SimCLR-ResNet inference via Grad-CAM (the incorrect examples). The left column: the original endoscopic images. The middle column: heatmaps based on the output of the feature extractor’s last layer of SimCLR-ResNet. The right column: the Grad-CAM heatmap covering the original images, in which the model mislocated the lesions

The outputs of the feature-extracted layers of supervised transfer learning and SimCLR were visualized by t-SNE, as shown in Fig. 5. The ternary samples showed better clustering through SSL in SimCLR than in supervised transfer learning. Furthermore, based on the outputs of SimCLR-ResNet, Grad-CAM was plotted, and an inferential explanation concerning the AI-inferred lesions is provided in Fig. 6 (the correct examples) and Fig. 7 (the erroneous examples).

## Discussion

In this study, we developed a series of SimCLR-based semi-supervised learning models to classify colorectal neoplasia based on the NICE classification. The ResNet-backboned SimCLR model showed an advantage over supervised transfer learning-based models and junior endoscopists, while it performed only slightly worse than senior endoscopists. The novel framework consists of (1) SSL on large unlabelled NBI-colonoscopic images and (2) fine-tuning on a few labelled images of colorectal neoplasia based on the NICE classification. Our study showed that, compared with traditional supervised learning, semi-supervised learning (which consists of SSL and fine-tuning) empowers AI models to achieve enhanced performances without relying solely on vast amounts of labelled data.

In the field of medical image analysis, deep learning has achieved remarkable performance in various competitive areas, such as pathology, radiology, and endoscopy [5, 22, 23]. However, the application of deep learning in low-resource settings faces challenges due to the scarcity of reliable labelled data [24]. SSL is now a novel solution for the successful use of various effective deep learning models [6]. These models are first pretrained without supervision using a source dataset and then fine-tuned for the target task [25]. Domain-specific SSL has proven to be effective at improving the medical image classification performance as compared to generic pretrained models [26]. Sun et al. used SimCLR as the backbone for a DL network to detect rib fractures from chest radiographs, showing superior detection sensitivity to traditional deep learning [8]. Ouyang et al. proposed a SimCLR-based self-supervised learning model to detect referable diabetic retinopathy, which overcame the training data insufficiency problem [27]. As demonstrated by the above studies, compared with traditional supervised learning, SSL can be used for computer-aided diagnosis of diseases that are difficult and time-consuming to label.

The application of SSL has shown notable promise in the detection, classification, and segmentation of medical images.

In the field of deep learning in gastrointestinal endoscopy, the current mainstream algorithm is still supervised learning [28–30]. Zhang et al. proposed a supervised-learning CNN model based on single-shot multibox detector architecture using 404 labelled endoscopic images with gastric polyps. The model realized real-time polyp detection with 50 frames per second and achieved 90.4% detection precision [31]. In recent years, with the introduction and maturation of self-supervised learning, it has gradually been applied in the field of endoscopy as well. We conducted a simple search for literature in the relevant field on PubMed, presented in Supplementary Table 5. In 2021, Du et al. [32] presented an SSL framework that employs an innovative module designed to generate efficient pairs for contrastive learning. By leveraging the similarity between images of the same lesion, this module enhanced the effectiveness of the contrastive learning process. Subsequently, an unsupervised approach was utilized to learn a visual feature representation that encapsulated the general features of esophageal endoscopic images. This representation was subsequently transferred to facilitate downstream esophageal disease classification tasks. Its results indicated that this framework achieved a classification accuracy surpassing that of other state-of-the-art semi-supervised methods. In 2023, in another study focused on *Helicobacter pylori* infection classification via blue laser endoscopic imaging, Jian et al. [33] proposed a self-supervised learning scheme consisting of an encoder and a prediction head. The encoder incorporated a backbone network, visual attention module, and feature fusion module to facilitate feature extraction through self-supervised contrastive learning. Once the encoder had been trained, the entire network was further fine-tuned using a small labelled image dataset. Through fivefold cross-validation experiments, it was observed that the proposed scheme achieved average *F*1-scores ranging from 0.885 to 0.915 for diagnosing *H. pylori* infection, outperforming existing methods. These diverse studies demonstrate that SSL techniques have shown potential in endoscopic diagnosis [6, 26]. The application of contrastive learning and self-supervised approaches has yielded notable improvements in accuracy and performance, addressing the challenge of limited labelled data.

In terms of the NICE classification, in 2022, Okamoto et al. [34] developed a supervised learning-based deep learning model for diagnosing colorectal lesions using the NICE classification. Coincidentally, ResNet was used as the backbone. Using a total of 4156 NBI images, the supervised learning model achieved a mean accuracy of 94.2%. In 2023, to address the challenge of limited labelled data availability, Krenzer et al. [30] proposed a few-shot learning (FSL) approach by creating an embedding space specifically tailored for colorectal lesions. FSL was designed to address the scarcity of labelled images [35]; specifically, in the transfer learning branch of FSL, the emphasis is on embedding learning. This process first involved training an embedding model that generates latent representations, allowing for task-specific notions of similarity between inputs to be quantified easily. Then, by structuring the latent space in such a way that samples from each class form distinct clusters, similarity metrics such as Euclidean or cosine distance can be used to determine the sample similarity and class affiliations. With this structural property, even with limited data available, simple class discrimination hypotheses can be constructed, such as *k*-nearest neighbor classification. In the study by Krenzer et al., the FSL-based model for NICE classification achieved an accuracy of 81.13%.

In this study, a SimCLR-based semi-supervised learning framework was developed to classify colorectal neoplasia using NBI colonoscopic images based on the NICE classification. Among the developed models, the ResNet-backboned SimCLR model exhibited better performance than the supervised transfer learning-based models. Given that the NICE classification was obtained through the consensus of three endoscopists, the classification performance was evaluated by junior and senior endoscopists independently. The semi-supervised learning model outperformed the endoscopist; however, it underperformed the senior endoscopist by 0.08% accuracy. In comparison with other computer-aided diagnosis methods for the NICE classification, our methods outperformed the FSL methods reported by Krenzer et al. [30] with almost 10% accuracy in datasets with scarce data. Moreover, the results of the proposed methods were similar to the results reported by Okamoto et al. [34], in which a total of 4156 NBI images were labelled for supervised learning. Finally, we visualized the advantage of SSL via t-SNE, which confirmed the improvement of the proposed framework.

There are several limitations in our study. First, despite quality review and selection of eligible images used for semi-supervised learning, the imperfections of the images are inevitable. It is possible that several NBI endoscopic images of a patient, captured from different angles, might have been included. Second, due to retrospective bias and the absence of clinical details and patients’ information in the public datasets, we failed to compare with datasets and provide more details. Third, the NICE classification system is determined by endoscopists under NBI endoscopy based on the color, microvascular structure, and surface pattern of the polyp. Its reliability is inferior to histopathology. Fourth, although we compared the classification performance of the supervised models and human endoscopists, the observational study of semi-supervised model-assisted compared with endoscopist-independent classification was not performed. Finally, further research and technology are required for real-time detection and classification to evaluate the performance of the semi-supervised model in clinical settings.

In this study, we presented a semi-supervised learning framework (SimCLR) for classifying colorectal neoplasia based on the NICE classification. Compared with traditional supervised learning, SSL empowers deep learning models to achieve improved performances with limited amounts of labelled endoscopic images.

## Supplementary Information

Below is the link to the electronic supplementary material.

Supplementary file1 (DOCX 619 KB)

## Data Availability

The code used to train SimCLR models can be found on an open-accessed website (https://osf.io/t3g8n).
