# Supplementary material for: A Semi-Supervised Learning Framework for Classifying Colorectal Neoplasia Based on the NICE Classification
Source: J Imaging Inform Med. 2024 Apr 23;37(5):2342–53. doi: 10.1007/s10278-024-01123-9 (PMC11522217; doi:10.1007/s10278-024-01123-9)
Supplement: Supplementary file 1 — Supplementary file1 (DOCX 619 KB) [file 10278_2024_1123_MOESM1_ESM.docx]

**Supplementary Introduction**

1) The detailed explanation of MCC and Cohen’s Kappa

MCC can be used as a measure of the quality od multiclass classification task, as shown in Schulza 2023[1]. It takes into account true and false positives and negatives and is particularly useful as it provides a high-quality measure of classification performance even with imbalanced class distributions.

MCC provides a single score that describes the overall quality of the classification, taking into account both the balance among classes and the importance of correctly classifying each class. A score near +1 indicates excellent performance, 0 indicates no better than random guessing, and -1 indicates total disagreement between prediction and observation[2, 3].

Cohen's Kappa measures the agreement between two raters who each classify N items into C mutually exclusive categories. Cohen's Kappa can be directly applicable to ternary classifications, to judge whether the prediction results are consistent with actual results in classification task, as shown in Lampe 2022[4]. The corresponding relationship between Kappa and consistency is as follows.

| Kappa value (x) | Consistency |
| --- | --- |
| x=-1 | Complete inconsistency |
| x=0 | Accidental consistency |
| x<0.20 | Slight consistency |
| 0.20≤x<0.40 | Fair consistency |
| 0.40≤x<0.60 | Moderate consistency |
| 0.60≤x<0.80 | Substantial consistency |
| 0.80≤x<1 | Perfect consistency |
| x=1 | Complete consistency |

2) The detailed explanation of t-SNE and Grad-CAM

The t-distributed Stochastic Neighbor Embedding (t-SNE), a non-linear, unsupervised algorithm for dimensionality reduction, is used to visualize high-dimensional data. t-SNE transforms high-dimensional data similarities into joint probabilities, then minimizes the difference between these probabilities in high-dimensional and low-dimensional spaces. This process helps to maintain the local structure of data. In the high-dimensional space, similarities are determined based on the probability that a point would choose another as its neighbor under a Gaussian distribution[5]. In the low-dimensional space, a student t-distribution calculates similarities. As shown in **Figure 5B**, the points corresponding to each color (labels of NICE Ⅰ, NICE Ⅱ, NICE Ⅲ,) were distinctly located in separate regions, indicating that the semi-supervised model had classified them accurately.

The Gradient-weighted Class Activation Mapping (Grad-CAM), an algorithm based on the gradient of an output class flowing into the final convolutional layer, is used to distinguish and highlight targeted regions that contribute to the network’s prediction by plotting a heatmap[6]. In the heatmap, the color is closer to red, the greater the weight of the lesion region determined by the model, whereas the color is closer to blue, the smaller the weight.

3) The reason for choosing above four backbones (VGG16, MobileNet, ResNet and Xception)

**VGG16:** Known for its indirect structure. All the convolutional layers use the same 3x3 convolution kernel. When the training dataset is small, it exhibits outs performance

**MobileNet:** Known for its efficiency, MobileNet serves as a benchmark for models aiming at applications where computational resources are limited, such as mobile devices or in-field diagnostic tools. Its performance sets a standard for speed and efficiency without significantly sacrificing accuracy.

**ResNet:** Known for its wide usage. With its innovative use of residual connections to enable training of very deep networks, ResNet represents a milestone in the development of CNNs. It has been widely applied in medical image processing.

**Xception:** Konow for its complexity.As a model that utilizes depth-wise separable convolutions for improved efficiency and performance, Xception It applies spatial convolution to each channel of the input image separately and then combines these convolution results through a 1x1 convolution.

**Supplementary Table1** NICE Type[7]

| NICE Type | | | | | |
| --- | --- | --- | --- | --- | --- |
| Type | Colorectal Endoscopic Findings | Color | Vessel pattern | Surface pattern | Invasion depth/ Pathology |
| Ⅰ | 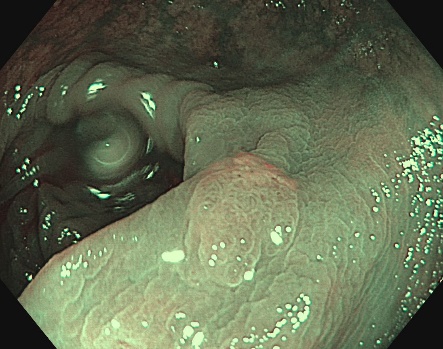 | Same or lighter than background | None, or isolated lacy vessels may vessels be present coursing across the lesion | Dark or white spots of uniform surface size, or homogeneous absence of pattern | HP/SSL |
| Ⅱ | 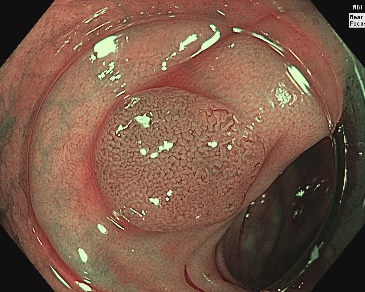 | Browner relative to background (verity color arises from vessels) | Brown vessels surrounding white structures | 0val, tubular or branched white structures surrounded by brown vessels | AD |
| Ⅲ | 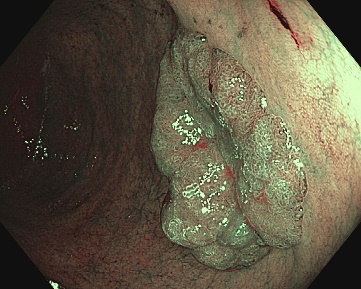 | Brown to dark brown relative to background sometimes patchy whiter areas | Has area(s) of disrupted or missing vessels | Amorphous or absent surface pattern | SM or deeper |

NICE: Narrow-band Imaging International Colorectal Endoscopic; HP: Hyperplastic Polys; SSL: Sessile Serrated Lesions; AD: Adenomas; SM: Submucosal invasive carcinomas

**Supplementary Table 2** Training parameters

|  | Semi-supervised learning | | Supervised transfer learning |
| --- | --- | --- | --- |
|  | Self-supervised learning | Fine-tuning |  |
| Batch size | 128 | 32 | 32 |
| Epoch number | 200 | 50 with early-stop | 50 with early-stop |
| Initial learning rate | 0.1 | 0.0001 | 0.0001 |
| Loss | Sparse Categorical Cross-entropy | Categorical Cross-entropy | Categorical Cross-entropy |
| Optimizer | SGD | Adam | Adam |
| Temperature | 0.1 | - | - |

**Supplementary Table 3**: Details of the two public datasets (PolypsSet[8] and PICCOLO[9])

| **Name** | **Publish year** | **Geographical distribution** | **Accessibility** | **Data type** | **Endoscopic type** | **Findings** | **Clinical details ^a^** |
| --- | --- | --- | --- | --- | --- | --- | --- |
| PolypsSet | 2021 | Combined with MICCAI 2017, CVC colon DB, GLRC dataset, and KUMC dataset | https://dataverse.harvard.edu/dataset.xhtml?persistentId=doi:10.7910/DVN/FCBUOR | Colon polyps | Colonoscopy | Polyps | No |
| PICCOLO | 2020 | Spain | https://www.biobancovasco.org/en/Sample-and-data-catalog/Databases/PD178-PICCOLO-EN.html c | Colon polyps | Colonoscopy | Polyps | Yes |

Continued

| **Name** | **Source of ground truth annotations** | | | **Endoscopy system brand** | | **Total images** | **Image pixels** | | **Imaging modality** | **Annotated/**  **Not annotated** | | **Classification of annotated images** |
| --- | --- | --- | --- | --- | --- | --- | --- | --- | --- | --- | --- | --- |
|  | **Ground truth ^b^** | **Content** | **Performed by** |  |  |  |  |  |  |  |  |  |
| PolypsSet | Bounding box | Polyps (hyperplastic and adenomatous polyps) | Expert endoscopists | NA | 155 videos (37899 images frames) | | | NA | NBI and WLI | 37899/0 | 87 videos of adenomatous polyps and 68 videos of hyperplastic | |
| PICCOLO | Binary mask | Polyps | Expert endoscopists | Olympus | 3433 images | | 854×480 or 1920×1080 | | NBI and WLI | 3433/ 0 | 2131 by WLI, 1302 by NBI | |

Continued

| **Name** | **Example paper** | **Application** | | **Model** | **Train** | **Test** | **Results (%)** | | | | | |
| --- | --- | --- | --- | --- | --- | --- | --- | --- | --- | --- | --- | --- |
|  |  |  |  |  |  |  | **Recall** | **Precision** | **F1-socre** | **Sensitivity** | **Specificity** | **Accuracy** |
| PolypsSet | Li et al. 2021 | Polyp detection | RefineDet | | 116 videos (28773 frames) | 22 videos (4872 frames) | 73.8 | 70.7 | 72.2 | NR | NR | NR |
| PICCOLO | Pacal I et al. 2022 | Polyp detection | | YOLO | PICOOLO | PICCOLO | 80.00 | 93.00 | 86.00 | NR | NR | NR |

For PolypsSet dataset, we selected 2000 unlabeled NBI images to train the self-supervised model. 2000 unlabeled images were extracted from 41 video sequences (26 of adenomatous polyps and 15 hyperplastic polyps).

For PICCOLO dataset, we selected and labeled 551 NBI images to fine-tune the semi-supervised model (NICE Ⅰ, n=219; NICE Ⅱ, n=221; NICE Ⅲ, n=111).

For Soochow/ Shanghai Jiao Tong University dataset, we selected and labeled 359 images to test the semi-supervised model (NICE Ⅰ, n=126; NICE Ⅱ, n=109; NICE Ⅲ, n=123). 359 labeled images were extracted from 88 hyperplastic polyps, 38 sessile serrated polyps, 91 adenomas, and 141 carcinomas.

The pathological type of polyps (adenomatous polyps, hyperplastic polyps, sessile serrated polyps and carcinomas) is diagnosed through histopathology, whereas the NICE classification is obtained by endoscopists based on features observed under NBI endoscopy.

**Supplementary Table 4** The characteristics of colorectal neoplasia in the test dataset (Soochow University and Shanghai Jiao Tong University)

|  |  | NICE I | NICE II | NICE III |
| --- | --- | --- | --- | --- |
| Number | | 126 | 109 | 123 |
| Lesion size (mm) | | 11.2 ± 4.3 | 12.6 ± 5.5 | 17.9 ± 6.6 |
| Histology | hyperplastic polyps | 88 | - | - |
|  | sessile serrated polyps | 38 | - | - |
|  | adenoma | - | 91 | - |
|  | carcinoma | - | 18 | 123 |

**Supplementary Table 5**: A review of relevant literatures regarding to the self-supervised learning on GI endoscopic image processing

| Title | Published year | | First Author | Country | Organ | Imaging modality | Cite |
| --- | --- | --- | --- | --- | --- | --- | --- |
| Improving the Classification Performance of Esophageal Disease on Small Dataset by Semi-supervised Efficient Contrastive Learning | | 2022 | Wenju Du | China | Esophagus | Gastroscope (WLI) | [10] |
| Self-supervised representation learning using feature pyramid siamese networks for colorectal polyp detection | | 2023 | Tianyuan Gan | China | Colorectum | Colonoscopy (WLI) | [11] |
| Improving image classification of gastrointestinal endoscopy using curriculum self‑supervised learning | | 2024 | Han Guo | America | Gastrointestinal tract | Gastrointestinal endoscopy (WLI) | [12] |
| DSMT-Net: Dual Self-Supervised Multi-Operator Transformation for Multi-Source Endoscopic Ultrasound Diagnosis | | 2024 | Jiajia Li | China | Pancreas | EUS | [13] |
| Time-based self-supervised learning for Wireless Capsule Endoscopy | | 2022 | Guillem Pascual | Spain | Small intestine and colon | WCE | [14] |
| Self-supervised out-of-distribution detection in wireless capsule endoscopy images | | 2023 | Arnau Quindós | Spain | Gastrointestinal tract | WCE | [15] |
| Improving Colonoscopy Lesion Classification Using Semi-Supervised Deep Learning | | 2021 | MAYANK GOLHAR | America | Colorectum | Colonoscopy (WLI and NBI) | [16] |

Continued

| Task | Images | | | | | Cite |  |
| --- | --- | --- | --- | --- | --- | --- | --- |
|  | Total | training | Validation/fine-tuning | testing | Image details |  | |
| Esophageal Disease detection | 23161 images | 16661 unlabeled esophageal images | 3500 expert-labeled esophageal images | 3000 images | Training and validation images were collected from West China Hospital of Sichuan University. Testing images were collected from Kvasir V2 dataset. The images were classified as seven categories: normal esophagus (NE), Oesophagitis (O), surgical scar (SC), esophageal varices (EV), esophageal submucous eminence (ESE), early esophageal cancer (EEC) and advanced esophageal cancer (AEC) | [10] | |
| Polyp detection | 263 videos | 103 unlabeled videos | 100 labeled videos | 60 videos | It comprises 40,266 frames of 200 polyps extracted from 160 colonoscopy videos, each with bounding boxes for every polyp. Additionally, it contains 103 videos, comprising 861,400 frames, which have only been simply annotated with video-level annotations that indicate the presence of polyps. | [11] | |
| Gastrointestinal findings classification | NR | 99417 unlabeled images | NR | NR | Images including pathological findings and anatomical landmarks in both the upper and lower GI tract | [12] | |
| PC and NPC classification | 11500 images | 8000 unlabeled images | 3000 labeled images | 500 labeled images | 3500 EUS enoted images, where the respective number of images captured by each device is recorded as 895, 990, 986, and 629. Moreover, our LEPset also has 8,000 EUS images without any classification annotation. | [13] | |
| Polyp detection | 40 videos with 1185033 frames | 95%/ 90%/ 80% unlabeled | 5%/ 10%/ 20% labeled | 2080 images | Training and validation images were from Generic WCE videos: only the small intestine and colon segments are used, selecting a total of 1,185,033 frames. Testing images were collected from Polyp WCE: 2080 containing polyps; Moreover, 1800 images from CAD-CAP WCE were used to test if the SSL process has captured enough rich information to avoid overfitting. | [14] | |
| ^*^ OOD images detection | 117 videos | 64 unlabeled videos | 10 labeled videos | 43 videos | The videos are comprised of 14 different classes, 9 of which refer to pathological categories we considered them as OOD images | [15] | |
| Polyp classification and domain adaptation testing | 132 videos | 112 unlabeled videos | NR | 20 labeled videos | the videos were categorized into two classes "neoplastic/precancerous” and “nonneoplastic”. 6649 frames were extracted from the videos | [16] | |

Continued

| Dataset | | | Cite |
| --- | --- | --- | --- |
| Name | Accessibility | Website |  |
| West China Hospital of Sichuan University; Kvasir V2 dataset | West China Hospital of Sichuan University: private dataset; Kvasir V2 dataset: public dataset | Kvasir V2:  https://www.kaggle.com/datasets/yasserhessein/the-kvasir-dataset | [10] |
| LDPolypVideo and CVC-VideoClinicDB | Both public datasets | LDPolypVideo:  https://github.com/dashishi/LDPolypVideo-Benchmark  CVC-VideoClinicDB: https://www.kaggle.com/datasets/balraj98/cvcclinicdb?select=metadata.csv | [11] |
| Hyper Kvasir | Public dataset | https://github.com/simula/hyper-kvasir | [12] |
| LEPset dataset | Public dataset | https://zenodo.org/records/8041285 | [13] |
| Generic WCE videos, Polyp WCE and CAD-CAP WCE | NR | Generic WCE videos: NR  Polyp WCE: NR  CAD-CAP: Unavailable | [14] |
| Kvasir-Capsule | Public dataset | https://github.com/simula/kvasir-capsule | [15] |
| Endoscopic center of Johns Hopkins Hospital | Private dataset | NR | [16] |

Continued

| Self-supervised learning model | | | Cite |
| --- | --- | --- | --- |
| Name | Self-supervised learning framework | Strategy for fine-tuning ^#^ |  |
| Semi-supervised efficient contrastive learning (SSECL) | Contrastive learning and generative learning | Feature-extraction strategy | [10] |
| Feature pyramid siamese networks (FPSiam) | Generative learning | End-to-end strategy | [11] |
| Curriculum Mixup (C-Mixup) | Contrastive learning and curriculum learning | NR | [12] |
| DSMT -Net | Contrastive learning and generative learning | End-to-end strategy | [13] |
| ResNet50-backboned SSL | Contrastive learning | Feature-extraction strategy | [14] |
| Out - distribution Detector for Neural Networks (ODIN) | Contrastive learning and clustering algorithm | NR | [15] |
| ResNet18-backboned SSL | Generative learning and jigsaw proxy task | End-to-end strategy | [16] |

Continued

| Metric and performance | | | | | | Cite |
| --- | --- | --- | --- | --- | --- | --- |
| SSL model | Metric and performance | Supervised model | | | Metric and performance |  |
| SSECL | Accuracy: 92.57%; Average Precision: 92.77%; Average Recall:92.57%; Average F1-score: 92.53% | | ResNet50 | Accuracy: 90.52%; Average Precision: 90.29%; Average Recall: 90.16%; Average F1-score: 90.29% | | [10] |
| FPSiam | When λ= 0.5, FPSiam method obtains the optimal mAP=24.3, AP_50_=49.6, AP_75_=20.5 | | Fast R-CNN | when λ= 0.5, Fast R-CNN method obtains the optimal mAP=22.0, AP_50_=50, AP_75_=15.9 | | [11] |
| C-Mixup | Accuracy 88.92%; Recall.75.0% | | NR | NR | | [12] |
| DSMT-Net | Accuracy: 0.877±0.005; Precision: 0.842±0.008; Recall: 0.801±0.013; F1 score: 0.822±0.009 | | ResNet101; DenseNet161 | ACC: 0.699; Precision: 0.538; Recall: 0.853; F1-score: 0.657  ACC: 0.761; Precision: 0.619; Recall: 0.842; F1-score: 0.706 | | [13] |
| ResNet50-backboned SSL | AUC: 90.00±2.09%; Sensitivity: 95% (80.16±6.97); 90%(86.31±6.20); 85% (92.09±4.63) | | ResNet50 | AUC: 92.94 ± 1.87%; Sensitivity: 95% (76.68 ± 4.93): 90%（82.86 ± 4.78）; 85%（88.53 ± 3.76） | | [14] |
| ODIN | Detection AU ROC 0.680 | | NR | NR | | [15] |
| ResNet18-backboned SSL | Polyp classification (with 25% labeled data): ACC:71.96%; Sensitivity:80%; F1-score: 81%  Domian adaptation: ACC:79.76%; Sensitivity:94.0%; F1-score: 79.76% | | End-to-end strategy | Polyp classification (with 25% labeled data): ACC:68.86%, Sensitivity:68%, F1-score: 77%  Domain adaptation: ACC:77.84%; Sensitivity:87.0%; F1-score: 77.84% | | [16] |

* OOD: out-of-distribution stands for lesions and anomalies which network have not seen before on endoscopic images

# In end-to-end fine-tuning, all the weights of the encoder and classifier are unfrozen and can be adjusted through optimization using supervised learning in the fine-tuning phase. In the feature-extraction strategy, the weights of the encoder are kept frozen to extract features as inputs to the downstream classifier.

WLI: White light imaging; EUS: Endoscopic ultrasonography; WCE: Wireless capsule endoscopy; NBI: Narrow band imaging; PC: Pancreatic cancer; NPC: non-pancreatic cancers; NR: Not report

PubMed was searched for relevant publications. Two independent authors used the following search query to perform a systematic search for relevant articles:

**((self-supervis*[Title/Abstract]) OR (self supervis*[Title/Abstract]) OR (contrastive learning[Title/Abstract]) OR (contrastive-learning[Title/Abstract]) OR (contrastive loss[Title/Abstract]) OR (contrastive loss[Title/Abstract]) OR (contrastive training[Title/Abstract]) OR (contrastive-training[Title/Abstract]) AND ((endoscop*[Text Word]) OR (colonoscop*[Text Word]) OR (gastroscop*[Text Word]) OR (Endoscopy[MeSH Terms]) OR (Colonoscopy[MeSH Terms]) OR (Gastroscopy[MeSH Terms]))**

After the initial search, we independently screened search results, and excluded several unqualified articles. The exclusion criteria were as follows: 1) duplicates; 2) unrelated to endoscopy; 3) unrelated to self-supervised learning. Moreover, articles focused on algorithmic innovation rather than specifically on gastrointestinal endoscopic image processing were also excluded, for example “Self-Supervised Lightweight Depth Estimation in Endoscopy Combining CNN and Transformer”. The dataset search and selection process flowchart were shown in **Supplementary Figure**.

It should be emphasized that we only search on PubMed, without extending to databases regarding to computer science paper. The reasons are as follows: 1). The sheer volume of relevant papers on computer science websites brings significant challenges in terms of search filtering and feature extraction, which we deemed unnecessary. 2). Papers on computer science websites are more likely to focus on algorithmic innovation rather than specifically on gastrointestinal endoscopic image processing.

Supplementary Figure 1 The method for endoscopist labeling


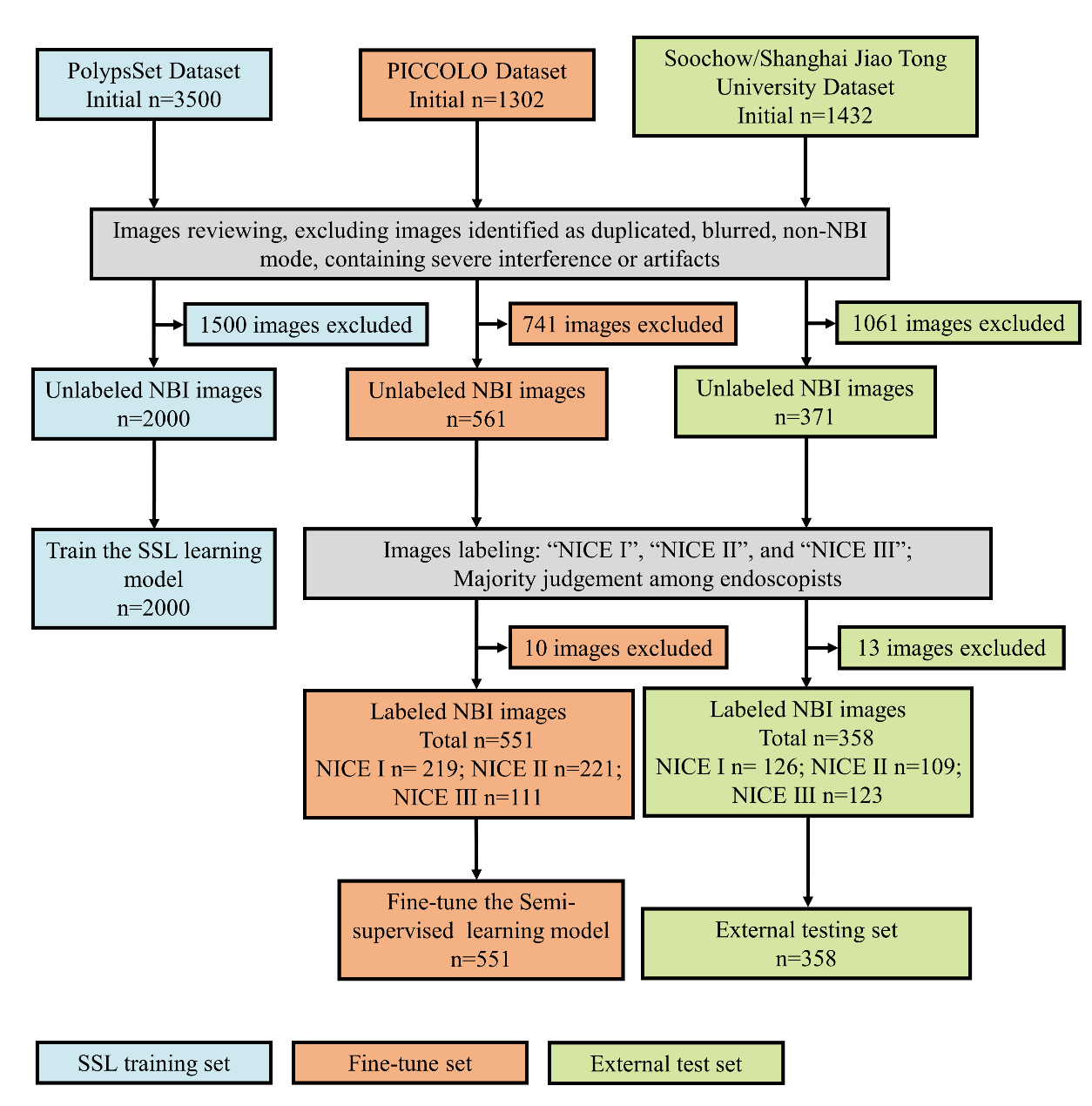


**Endoscopists review and labeling**

Three endoscopists reviewed and provided judgments on the NBI endoscopic images, the endoscopists were all fellowship-trained in endoscopy with more than ten years of endoscopic experience. During the review process, if any endoscopist identified an image as duplicated, blurred, non-NBI mode, or containing severe interference or artifacts, the image is excluded. After reviewing eligible images, each endoscopist independently labeled each image from the PICCOLO dataset and Soochow University/Shanghai Jiao Tong University dataset in order to classify it into one of three types: “NICE Ⅰ”, “NICE Ⅱ”, and “NICE Ⅲ”. The majority judgement among the three endoscopists was taken as the “ground label” for each image. Moreover, to ensure the accuracy of the labels, three endoscopists were provided with ample time (more than 1 week). A custom web interface was constructed to allow the reviewers to window, zoom, manipulate, and categorize each image.

**Reference**

1. Schulz D, et al.: A deep learning model enables accurate prediction and quantification of pulmonary edema from chest X-rays. Crit Care 27:201, 2023

2. Sokolova M, Lapalme G: A systematic analysis of performance measures for classification tasks. Information Processing & Management 45:427-437, 2009

3. Chicco D, Jurman G: The advantages of the Matthews correlation coefficient (MCC) over F1 score and accuracy in binary classification evaluation. BMC Genomics 21:6, 2020

4. Lampe L, et al.: Comparative analysis of machine learning algorithms for multi-syndrome classification of neurodegenerative syndromes. Alzheimers Res Ther 14:62, 2022

5. Liu J, Vinck M: Improved visualization of high-dimensional data using the distance-of-distance transformation. PLoS Comput Biol 18:e1010764, 2022

6. Selvaraju RR, Cogswell M, Das A, Vedantam R, Parikh D, Batra D: Grad-CAM: Visual Explanations from Deep Networks via Gradient-Based Localization. International Journal of Computer Vision 128:336-359, 2020

7. Tanaka S, Sano Y: Aim to unify the narrow band imaging (NBI) magnifying classification for colorectal tumors: current status in Japan from a summary of the consensus symposium in the 79th Annual Meeting of the Japan Gastroenterological Endoscopy Society. Dig Endosc 23 Suppl 1:131-139, 2011

8. Li K, et al.: Colonoscopy polyp detection and classification: Dataset creation and comparative evaluations. PLoS One 16:e0255809, 2021

9. Sanchez-Peralta LF, et al.: PICCOLO White-Light and Narrow-Band Imaging Colonoscopic Dataset: A Performance Comparative of Models and Datasets. Applied Sciences 10:8501, 2020

10. Du W, et al.: Improving the Classification Performance of Esophageal Disease on Small Dataset by Semi-supervised Efficient Contrastive Learning. J Med Syst 46:4, 2021

11. Gan T, Jin Z, Yu L, Liang X, Zhang H, Ye X: Self-supervised representation learning using feature pyramid siamese networks for colorectal polyp detection. Sci Rep 13:21655, 2023

12. Guo H, Somayajula SA, Hosseini R, Xie P: Improving image classification of gastrointestinal endoscopy using curriculum self-supervised learning. Sci Rep 14:6100, 2024

13. Li J, et al.: DSMT-Net: Dual Self-Supervised Multi-Operator Transformation for Multi-Source Endoscopic Ultrasound Diagnosis. IEEE Trans Med Imaging 43:64-75, 2024

14. Pascual G, Laiz P, García A, Wenzek H, Vitrià J, Seguí S: Time-based self-supervised learning for Wireless Capsule Endoscopy. Comput Biol Med 146:105631, 2022

15. Quindós A, Laiz P, Vitrià J, Seguí S: Self-supervised out-of-distribution detection in wireless capsule endoscopy images. Artif Intell Med 143:102606, 2023

16. Golhar M, Bobrow TL, Khoshknab MP, Jit S, Ngamruengphong S, Durr NJ: Improving Colonoscopy Lesion Classification Using Semi-Supervised Deep Learning. IEEE Access 9:631-640, 2021
